# Glycerol 3-phosphate dehydrogenases (1 and 2) in cancer and other diseases

**DOI:** 10.1038/s12276-024-01222-1

**Published:** 2024-05-01

**Authors:** Sehyun Oh, Xuan Linh Mai, Jiwoo Kim, Arvie Camille V. de Guzman, Ji Yun Lee, Sunghyouk Park

**Affiliations:** 1 College of Pharmacy, Natural Products Research Institute, Seoul National University, Seoul, 08826 Korea; 2 Department of Cancer Biology, Dana-Farber Cancer Institute, Harvard Medical School, Boston, MA 02215 USA; 3 School of Biological Sciences, Seoul National University, Seoul, 08826 Korea

**Keywords:** Metabolic disorders, Mechanisms of disease

## Abstract

The glycerol 3-phosphate shuttle (GPS) is composed of two different enzymes: cytosolic NAD^+^-linked glycerol 3-phosphate dehydrogenase 1 (GPD1) and mitochondrial FAD-linked glycerol 3-phosphate dehydrogenase 2 (GPD2). These two enzymes work together to act as an NADH shuttle for mitochondrial bioenergetics and function as an important bridge between glucose and lipid metabolism. Since these genes were discovered in the 1960s, their abnormal expression has been described in various metabolic diseases and tumors. Nevertheless, it took a long time until scientists could investigate the causal relationship of these enzymes in those pathophysiological conditions. To date, numerous studies have explored the involvement and mechanisms of GPD1 and GPD2 in cancer and other diseases, encompassing reports of controversial and non-conventional mechanisms. In this review, we summarize and update current knowledge regarding the functions and effects of GPS to provide an overview of how the enzymes influence disease conditions. The potential and challenges of developing therapeutic strategies targeting these enzymes are also discussed.

## Unveiling GPD1 and GPD2: key players in cancer metabolism

Glycerol 3-phosphate dehydrogenases (GPDs, enzymes that help in the body’s metabolism) are important for converting carbohydrates to fats. Their role in diseases like cancer is unclear. Studies show that two types of GPDs, GPD1 and GPD2, can affect cancer growth differently. GPD1 usually stops cancer cell growth, while GPD2 often encourages it. This is believed to be due to their impact on different metabolic processes in the cell. The research suggests that focusing on these enzymes could be a new way to treat cancer. However, more studies are needed to fully understand how this works and to create effective treatments. This research could greatly impact future cancer treatments. This summary was initially drafted using artificial intelligence, then revised and fact-checked by the author.

## Introduction

**Fig. 1:**
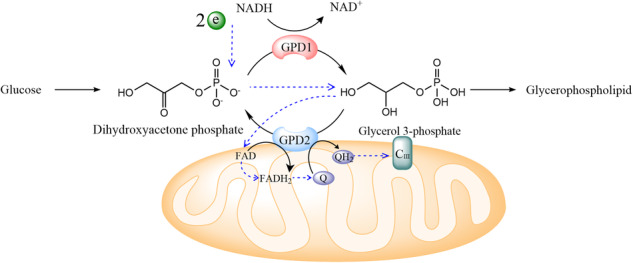
Enzymatic reactions mediated by GPD1 and GPD2. GPD1, located in the cytosol, reduces the glycolytic intermediate dihydroxyacetone phosphate (DHAP) to glycerol 3-phosphate (G3P) using two electrons from NADH, thus generating NAD^+^. On the outer surface of the IMM, GPD2 oxidizes G3P back to DHAP by reducing FAD to FADH_2_ (dehydrogenase activity). It also mediates electron transfer from FADH_2_ to ubiquinone (Q) (oxidoreductase activity). The blue dashed arrows denote the flow of electrons (depicted as a green ball with “e”).

The first observation of glycerol 3-phosphate (G3P) oxidation was reported in 1919^1^. With subsequent studies, the presence of two G3P dehydrogenases (GPDs) and their reactions, constituting the G3P shuttle (GPS), were discovered in the flight muscles by the 1960s^2,3^. Alongside the malate-aspartate shuttle (MAS), the GPS is one of the two mitochondrial NADH shuttles that transports reducing equivalents from cytosolic NADH across the inner mitochondrial membrane (IMM) to the mitochondrial electron transport chain (ETC)^4^. The two GPDs of GPS are NADH-dependent, cytosolic G3P dehydrogenase (GPD1) and FAD-dependent, mitochondrial G3P dehydrogenase (GPD2). GPD1 catalyzes the conversion of the glycolytic intermediate dihydroxyacetone phosphate (DHAP) to G3P using NADH. On the other hand, GPD2, which is located on the outer surface of the IMM, catalyzes the reverse reaction: it converts G3P back to DHAP with the reduction of FAD to FADH_2_, consequently transferring electrons to coenzyme Q (CoQ) in the ETC (Fig. 1)^2,5^.

Because the activity of GPD1 is lower than that of lactate dehydrogenase in mammalian tissues^6^ and because GPS is less efficient than MAS in terms of ATP generation (1.5 ATP per NADH for GPS and 2.5 for MAS), the functional roles of GPS were not highlighted until the 1970s, when it was discovered that mammalian brown adipose tissue (BAT) exhibits high activities of GPD1 and GPD2^7,8^. Prominent studies by Albert Lehninger suggested that the MAS accounts for all the NADH transferred to mitochondria generated from glucose to pyruvate catabolism (glycolysis) in tumor cells, might also have led to less interest in the GPS^9,10^. Currently, more than four decades of research have shown that GPS and its component enzymes work as central hubs in relation to glucose metabolism, lipid metabolism, and mitochondrial respiration. The overall importance of these enzymes was further evidenced by neonatal lethality and substantial metabolic alterations in mice lacking both GPD1 and GPD2^11^. In this review, we combined several critical studies on the roles of the GPS proteins GPD1 and GPD2 to summarize the current understanding of the metabolic and functional impacts of GPS, particularly focusing on their involvement in diseases such as cancer. A list of the involvements of GPDs in various non-cancerous diseases is provided in Table 1. Due to page limitations, we might not have cited all the relevant studies, and due credit should be given to those studies. For readers seeking a detailed historical review of GPD2 up to 2013, we recommend an excellent review by Tomas Mráček and coworkers^12^.

**Table 1:** The roles and mechanisms of GPDs in diverse pathologies.

Gene	Disease	Tissue	Implication	Mechanism	Ref.
GPD1	Obesity	Adipose	Pro-obesity	Generates G3P leading to an increase in TG accumulation	^21–23^
		Muscle	Fatty acid oxidation in skeletal muscle	Possibly regulated by EID1	^33–35^
	Transient infantile hypertriglyceridemia	Liver	Mutation of *GPD1* associated with TG secretion	Regulation of DHAP and fatty acid oxidation	^24–28^
	Neuroinflammation	Brain	Low GPD1 activity in brain contributes to neuronal susceptibility to mitochondrial complex I dysfunction	GPD1 overexpression regenerates NAD^+^ and enhances G3P synthesis in complex I-compromised conditions	^38^
	N/A	In vitro cell line	Antioxidant	Unknown	^36,37^
GPD1L	Brugada syndrome	Heart	*GPD1L* mutation associated with Brugada syndrome and cardiac sudden death	Association with SCN5A, altering inward sodium current in the heart	^42,43^
GPD2	Obesity	Adipose	BAT thermogenesis	Unknown	^73^
		Muscle	Muscle regeneration and myoblast differentiation	Increases NAD^+^/NADH and activates AMPK/PGC1a resulting in mitochondrial biogenesis	^77^
	Diabetes	Pancreas	Implicates in glucose-mediated insulin secretion	Altering glycolysis activity through NAD^+^/NADH shuttling	^81–83^
		Kidney	Protects podocytes	Inhibits RAGE pathway, enhancing mitochondrial biogenesis/metabolism and lowering ROS	^114^
	Steatosis	Liver	GPD2 loss leads to ER-stress-induced steatosis	Induction of ubiquitin-mediated degradation of cyclophilin D that activates PTP, altering mitochondrial calcium release	^78^
	N/A	Brain	Suggested to be involved in neurotransmission	Unknown	^95^
	Inflammatory diseases	Immune system	T-cell activation	Hyper-reduction of ubiquinone, generation of ROS during TCR signaling	^103^
			LPS tolerance of macrophages	Boosting glucose oxidation to support acetyl-CoA for histone acetylation of inflammatory genes upon acute LPS stimulation. Induce RET and reduction in oxidative metabolism; reverse histone acetylation and macrophage activation upon prolonged LPS stimulation.	^104^
	Ischemic disease	Heart	Responsible for cell death during IRI	ROS release	^111,112^
			Protection against MI	Calcium influx in MI activates GPD2 facilitating ATP synthesis from glycerol as an adaptation to the limited oxygen supply	^113^
	N/A	Sperm	Acrosome reaction	ROS generation in spermatozoa	^107^

## Glycerol 3-phosphate dehydrogenases: individual enzymes and involvement in metabolic diseases

### Glycerol 3-phosphate dehydrogenase 1 (GPD1)

#### Gene and protein structure

The *GPD1* gene is 7306 bp in length and is located on human chr12q13.12. It includes eight exons and seven introns and encodes a protein consisting of 349 amino acids. It does not seem to be an essential gene for life, as orthologs have not been identified in many prokaryotes or even in some eukaryotes. The three-dimensional structure of human GPD1 was solved with X-ray crystallography^13^, revealing two distinct regions: the C-terminal domain, which has several helical structures where the substrate DHAP binds; and the N-terminal domain, which contains a β-sheet core for NADH binding. This study also revealed the substrate binding and catalytic mechanism, indicating that Arg269 and Lys120 contribute to the binding of the substrate DHAP, and Lys204 polarizes the carboxyl group of DHAP for hydride attack from NADH.

#### Regulation

The regulation of GPD1 activity seems to occur mostly at the transcriptional level. *GPD1* has been reported to be a direct target of peroxisome proliferator-activated receptor alpha (PPARα) in the liver and PPARγ in adipose tissues^14^. The upregulation of *GPD1* by PPARγ and the expected increase in G3P were proposed to be related to the enhanced lipid storage and insulin-stimulated fatty acid uptake in adipose tissues induced by a thiazolidinedione, a PPARγ agonist used for treating type 2 diabetes^15^. Additionally, androgen-mediated regulation of *GPD1* for hepatic gluconeogenesis has been reported^16^. Another example of transcriptional regulation involves mutual positive regulation between hypoxia-inducible factor 1-alpha (HIF1α) and GPD1 in clear cell renal cell carcinoma (ccRCC)^17^.

Non-transcriptional regulation was also reported, whereby GPD1 expression was markedly elevated during adipogenesis in mice due to histone H3 lysine K4 (H3K4) methylation^18,19^. In glycerol-producing yeast, Gpd1 and Gpd2, both of which are homologous to mammalian GPD1, were shown to be inhibited by phosphorylation under different metabolic conditions by AMP-activated protein kinase (AMPK) and target of rapamycin complex 2^20^. However, mammalian cells do not typically operate glycerol fermentation, and similar phosphorylation in higher animals has not been demonstrated. Therefore, more studies are needed to determine the existence and functional relevance of posttranslational modifications.

#### Functions and implications in non-cancer diseases

As GPD1 generates G3P, which connects carbohydrate and lipid metabolism and is involved in NADH/NAD^+^ recycling, abnormal activity of GPD1 is expected to cause metabolic diseases. There is evidence showing that GPD1 has pro-obesity effects. Enhanced GPD1 activity has been observed in morbidly obese patients, and correlations between GPD1 expression and obesity, body mass index (BMI), and fat mass were found^21,22^. As fatty acid synthase (FAS) and ATP citrate lyase (ACLY) activities were lower in obese patients’ adipose tissues, GPD1-generated G3P was suggested to be the driving force for triglyceride (TG) accumulation. In line with this, GPD1 expression in the skeletal muscle of obese individuals was high, and it could be reversed after weight loss surgery that improved BMI and insulin resistance, and was associated with TG accumulation^23^. On the other hand, other studies have shown that GPD1 plays a role in protection against hyperlipidemia or liver steatosis. Basel-Vanagaite et al. demonstrated that a truncation mutation of *GPD1* is associated with increased secretion of TGs, leading to hypertriglyceridemia, which was supported by cell-based experiments^24^. Subsequently, several clinical mutations in *GPD1* have been reported with consistent phenotypes, such as hepatomegaly, steatosis, or hypertriglyceridemia, often in infants^25–28^. For example, a compound heterozygous mutation in *GPD1* leading to the absence of the protein in the liver of a female infant caused hepatomegaly, steatosis, and hypertriglyceridemia^25^. This study also suggested that GPD1 deficiency leads to lipid accumulation via two mechanisms: DHAP accumulation or decreased fatty acid oxidation. Given the pro-obesity or anti-lipid accumulation activity of GPD1, as stated above, it is difficult to propose a unified idea on the role of GPD1 in human obesity.

In comparison, mice lacking GPD1 exhibited the most extensive metabolite changes in skeletal muscle, with many minor changes in the liver and kidney (limited to those in which GAPDH is upregulated during glycolysis)^29,30^. Notably, these mice are not laboratory-generated *GPD1* knockout (KO) mice but are a subline of BALB/c (BALB/cHeA) mice with a natural *GPD1* mutation leading to the loss of GPD1^31,32^. Despite the metabolite alterations (in G3P, DHAP, and the lactate/pyruvate ratio), the exercise tolerance and pancreatic islet function of the mutant mice were normal. On the other hand, later studies revealed that mice without GPD1 had higher fatty acid oxidation in skeletal muscle, leading to higher exercise endurance and less weight gain^33,34^, possibly regulated by EP300 interacting inhibitor of differentiation 1 (EID1)^35^. Although other roles of GPD1, such as protection from cell death upon treatment with oxidants, have been reported with a cell line^36,37^, confirmation is necessary in in vivo systems. Therefore, the exact roles of GPD1 in obesity, muscle function, and protection against oxidants have not been well established. In the opposite context of the GPD1 level, the overexpression of GPD1 partially rescued survival, the alpha-hydroxybutyrate level (representing the NADH/NAD^+^ ratio), motor function, and neuroinflammation in the Ndufs4^-/-^ mouse modeling human neuropathies^38^. As the study focused on the roles of GPD1 in complex I (CI)-compromised conditions, it remains unclear whether those functions are applicable to normal conditions.

It may be worthwhile to comment on a related protein called GPD1L, whose gene was first discovered in 2002^39^. This gene is located on human chr3p22.3, very close to *SCN5A*, whose mutation is associated with Brugada syndrome, an autosomal-dominant form of idiopathic arrhythmia potentially leading to sudden death^40^. The protein encoded by *GPD1L* has 84% amino acid homology to GPD1 and was proposed to be associated with the plasma membrane^41^. It is possible that, through its association with SCN5A, GPD1L is also associated with Brugada syndrome and sudden cardiac death^42^. A *GPD1L* mutation appears to reduce its association with SCN5A, a sodium channel, consequently lowering the inward sodium current in the heart^42,43^. Additionally, GPD1L expression has been shown to be downregulated in certain types of cancer, such as head and neck squamous cell carcinoma and renal cell carcinoma^39,44^. Given that *GPD1* mutation is associated with transient infantile hypertriglyceridemia, the disease association and intracellular localization seem different from those of GPD1L.

Overall, there seem to be at least three variables to consider in understanding GPD1 functions: species, tissues, and compensation. As human *GPD1* mutations were identified more recently than mutations in mice, future studies should devote more attention to the differences between the species. In mouse experiments, GPD1-deficient mice are not specifically generated for *GPD1* deletion; therefore, they might have other unknown abnormalities. For tissues, mice deficient in GPD1 exhibited very different metabolite changes according to tissues. This might be related to tissue-specific compensation, e.g., less compensation by glycerol kinase in muscle than in liver or kidney. Compensation for GPD1 activity by GPD1L might also be possible, but the *GPD1* gene is responsible for almost all glycerophosphate dehydrogenase activity in all adult tissues, with the liver and possibly the kidney depending entirely on *GPD1*^32^. Therefore, GPD1L compensation may need to be further tested experimentally.

### Glycerol 3-phosphate dehydrogenase 2 (GPD2)

#### Gene and protein structure

The *GPD2* gene on human chr2q24.1 spans a 150,025-bp region, including 17 exons and 16 introns, and its coding sequence encodes 727 amino acid residues. Unlike GPD1, GPD2 is a conserved protein with an ortholog even in *E. coli*. The bacterial version, GlpD, is associated with the bacterial cell membrane, corresponding to the mitochondrial membrane in eukaryotes, and is devoid of ~100 residues homologous to calmodulin at the C-terminus^45^. GlpO is another ortholog of GPD2 in bacteria. GlpO is a soluble, cytosolic, and FAD-linked glycerol phosphate oxidase that can reduce O_2_ to H_2_O_2_ in some heme-deficient bacteria, such as *Enterococcus casseliflavus*^46,47^. To date, the three-dimensional structure of human GPD2 has not been determined, and much structural and mechanistic information has been obtained using the crystal structure of detergent-solubilized *E. coli* GlpD^48^, which has ~45% sequence similarity (30% identity) with the mammalian enzyme. Bacterial GlpD has two distinct regions with opposite electrostatic potentials. The C-terminal domain, which has a negative electrostatic surface, assists in the recognition of the phosphate group. The N-terminal domain consists of an FAD- and substrate-binding domain and has overall positive potential, enabling its interaction with negatively charged phospholipids of the membrane. Notably, the base region of the FAD-binding domain was suggested to be embedded into the lipid bilayer by ~12–15 Å, based on its observed interaction with detergent molecules. Interestingly, this region also contains a putative ubiquinone docking site comprising a hydrophobic plateau, providing insights into how electrons are transferred to CoQ. Although the bacterial enzyme structure provides valuable information on substrate binding, lipid-enzyme interactions and a possible electron transfer mechanism have yet to be confirmed in the context of mammalian enzymes. The dimeric form of bacterial GlpD in the crystal structure is consistent with a suggested functional dimer for GPD2^49^, but this could still be due to crystal packing. A recent study of the purification of active GPD2 suggested a monomeric status^50^; therefore, the true oligomeric status may require future studies.

#### Regulation

Compared to *GPD1*, which is expressed in almost all tissues^29^, the expression of *GPD2* is quite selective and is thus regulated by more factors. However, transcriptional regulation is the primary mechanism, and *GPD2* is regulated by at least three different promoters, namely, promoters A, B, and C^51–53^. These promoters are associated with different 1st exons in tissue-specific manners (e.g., promoter A in the brain, promoter B in all tissues, and promoter C in the testis), which might partially explain the highly differential expression of GPD2 across tissues. Interestingly, ubiquitous promoter B, which is the only promoter regulated by thyroid hormone in the liver^53^, does not respond to thyroid hormone in tissues with high GPD2 expression (e.g., brown adipose tissue, brain, and testis), suggesting that some tissue-dependent factors, such as Sp1, are involved in regulation by promoter B^54,55^. Additional complexity in promoter B regulation was reported wherein the promoter B is sensitive to thyroid hormone in rats^54^ but not in humans^56^, thus suggesting species-differential regulation. Although it is not exactly a transcriptional regulation, miR-1 and miR-206 have been shown to regulate the 3′-UTR of *GPD2* as a downstream mediator of NRF2 signaling^57^.

Other mechanisms, including cofactors or posttranslational modifications, can regulate GPD2. Ca^2+^ is an important cofactor, possibly acting through the C-terminal putative calmodulin-like domain that is absent in the *E. coli* GlpD structure^48^. As substrate binding occurs in a different region, it may be an allosteric regulator that enhances GPD2 activity by lowering the K_m_ for the substrate^58–60^. A recent study revealed the functional relevance of the Ca^2+^-mediated regulation of the GPS in terms of ATP generation for the electrical activity of hippocampal neurons^61^. This mitochondrial Ca^2+^-regulated GPS activity, which involved GPD2, was suggested to act as a backup system for more prominent Ca^2+^-dependent ATP generation systems, such as the MAS^62^ and mitochondrial calcium uniporter-driven activation of Ca^2+^-sensitive TCA enzymes^63^. Other important allosteric regulators of GPD2 are free fatty acids (FFAs) and their acyl-CoA esters. Even in the presence of Ca^2+^, palmitoyl-CoA inhibited the activity of GPD2 at a very low concentration by decreasing G3P availability in a competitive manner^64^. Inhibition by FFAs is more complex in that oleate inhibited oxidoreductase activity, which transfers electrons from GPD2 to CoQ, but not dehydrogenase activity, which oxidizes G3P in a non-competitive and BSA-relievable way^65^. Other studies have shown that the inhibitory effects of FFAs might occur by altering membrane microviscosity or composition^66,67^. It might be suggested that a high concentration of FFAs may shift metabolism toward glycerolipid synthesis through a dual mechanism: 1) serving as a substrate for esterification or 2) increasing G3P by inhibiting GPD2. Not only small molecules but also protein factors have been reported to regulate GPD2. GCN5L1, known as a mitochondrial acetyltransferase, regulates GPD2 activity by protein interactions to support gluconeogenesis in the liver, as demonstrated by respiration measurements and coimmunoprecipitation^68^.

Post-translational or covalent modification of GPD2 also controls its activity. Phosphorylation at T10 by protein kinase delta enhances its substrate affinity in glioma cells^69^. Although some studies have proposed that GPD2 has catalytically important SH- groups^70,71^, it is only modestly inhibited by thiol-targeting chemicals. Instead, its activity is more profoundly inhibited by chemicals that modify tyrosyl, lysyl, or histidyl side chains^71^. Not only modification but also removal of residues affects GPD2 activity. GPD2 in prostate cancer cells forms functional dimers only after the removal of the N-terminal 42 residues^49^. This processing seems to be mediated by the inner membrane protease IMMP2L, which, unlike GPD2, does not exhibit a biased distribution across tissues. Nevertheless, it is unclear whether the N-terminal sequence is required for the enzyme activity per se or whether it is just for the mitochondrial targeting and inner membrane localization of GPD2. Additionally, the tissue specificity of this process needs to be further studied, especially considering the phosphorylation of T10 and the subsequent activation of GPD2 in glioma cells, as stated above^69^.

#### Functions and implications in non-cancer diseases

The high activity of GPD2 in BAT^51^, along with the fact that GPD2 was originally discovered in the flight muscles of *Drosophila*^2^, suggest that GPD2 plays a significant role in energy production and utilization as an important factor for thermogenesis. In one study, *GPD2* KO mice did not exhibit hypothermia or defective gross thermogenicity, and the temperature increased normally in response to thyroid hormone treatment^72^. However, a later study employing another breed of *GPD2* KO mice revealed that there was a small but noticeable decrease in energy expenditure, despite the increase in the serum thyroid hormone level^73^. The authors suggested that GPD2 is important in thyroid hormone-mediated thermogenesis and further showed that the absence of GPD2 caused a state of sustained cold stress that incurred compensatory heat generation through BAT and skeletal muscle uncoupling protein 3 expression. The apparent discrepancy between the two studies might be reconciled considering the supraphysiological thyroid hormone used in the first study, which involves multiple thermogenic mechanisms not dependent upon GPD2 and normal gross thermogenesis upon the deletion of possible thermogenic genes^74,75^. The KO mice in the second study were further characterized as having a “thrifty” phenotype by the same group^76^.

A common phenotype between the above two breeds is a small but significant weight loss (~20% in the first and ~5% in the second). However, another *GPD2* KO breed did not experience weight loss, but defects in muscle regeneration after injury were detected^77^. Although muscle development and myofibril size remained normal with no gross histological defects, *GPD2* KO mice exhibited impaired muscle regeneration and myoblast differentiation, which may have implications for decreasing muscle mass in obese and diabetic patients. Mechanistically, GPD2 increased the NAD^+^/NADH ratio accompanied by activation of the AMPK/PGC1α axis, ultimately resulting in mitochondrial biogenesis. An earlier *GPD2* KO breed exhibited higher G3P and lactate/pyruvate ratios in muscle^76^, suggesting increased glycolysis; future studies should perform a comparison with the GPD2 regulation of muscle glycolysis. Additionally, despite the decrease in white adipose tissue (WAT) in an earlier KO breed^72^, the reported inconsistency in weight loss makes it difficult to determine the effects of GPD2 on WAT adiposity, weight changes, or obesity at this point. Interestingly, liver-specific GPD2 loss caused ER stress-induced liver steatosis through increased release of mitochondrial calcium via the permeability transition pore (PTP)^78^. A mechanistic investigation revealed that GPD2 induced the ubiquitin-mediated degradation of cyclophilin D that activates PTP. Therefore, the regulation of lipids by GPD2 may also be tissue specific, employing different pathways.

As the NAD^+^/NADH ratio is directly related to the function of GPD2 as one of the two NADH shuttle systems, its physiological involvement has been studied where the GPD2 level is high. One such tissue is pancreatic beta cells, which secrete insulin with high aerobic glycolysis activity^79,80^. As early as the 1980s, GPD2-mediated bioenergetic metabolism was implicated in glucose-mediated insulin secretion, linking GPD2 to diabetes^81–83^. Later, a study with pancreatic islets from *GPD2* KO mice revealed that blocking both NADH shuttles (GPS and MAS) is required for the inhibition of glucose-induced insulin secretion^84^, revealing the redundant roles of GPS and MAS. Additionally, overexpression of GPD2 could not rescue the impairment in glucose-induced insulin secretion in GPD2-low GK rats^85^ or GPD2-low rodent cells^86^. Compared to these genetic studies performed in rodent systems, *GPD2* mutations in human patients were linked to type 2 diabetes^87,88^, and autoantibodies against GPD2 were detected in insulin-dependent diabetic patients^89,90^. Therefore, there might be differences between rodent and human systems, and it will be interesting to determine whether there are coexisting mutations or malfunctions in MAS in GPD-mutant human patients.

Another tissue with relatively high GPD2 activity is the brain. The absence of an increase in lactate in brains with ARALAR deficiency (a component of the MAS)^91^, in contrast to the increase in lactate in the brain in most cases of mitochondrial dysfunction^92–94^, was attributed to GPS being a major NADH shuttle in astrocytes, not in neurons. Another study of the brain showed that high GPD2 activity was present in areas with high synaptic density in the mouse brain, such as the hippocampal stratum oriens, suggesting a role for GPD2 in neurotransmission^95^. However, there is controversy regarding the presence of GPD1 or GPD2 in different types of brain cells. For example, GPD1 is selectively expressed in oligodendroglial cells^96,97^, in contrast to the selective presence of GPD2 in neurons, making the role of GPS as a NADH shuttling machinery irrelevant in the brain^95^. Nevertheless, the GPS might function with another GPD1 isotype, GPD1L, and the actual activities of cytosolic and mitochondrial GPD were observed at similar levels^91^. Therefore, histological (antibody staining), pharmacological (inhibitors), and biochemical (enzyme activity) experiments have provided somewhat discordant views on the roles of GPS in different brain cell types, and studies incorporating different approaches in the same setting should be performed to obtain better insight. Apart from GPS activity, targeting GPD2 with metformin in brain abnormalities has been studied^98,99^, but the effect may not be specifically due to GPD2 inhibition, considering the various effects of metformin.

NAD^+^/NADH shuttling by GPD2 transfers reducing equivalents to the mitochondrial ETC, and overactivation of this process can cause reactive oxygen species (ROS) generation through reverse electron transport (RET)^100–102^. Interestingly, this phenomenon has been found in immune cell modulation. During T-cell activation, T-cell receptor (TCR) signaling shifted glycolytic flux from the GAPDH direction to the GPD1 direction using DHAP as the substrate^103^. The subsequent activation of GPS led to RET through the hyperreduction of ubiquinone and the generation of ROS at CI. This was followed by ROS-induced NF-kB-dependent gene expression, such as that of IL-2 and IL-8. A slightly more intricate role of GPD2-mediated RET was also reported in macrophages under LPS stimulation^104^. The enhanced activity of GPD2 was responsible for boosting glucose oxidation to support acetyl-CoA production and thus to provide materials for histone acetylation for the induction of pro-inflammatory genes in the acute phase. However, prolonged exposure to LPS and long-lasting GPD2 activation led to RET and a reduction in oxidative metabolism, reversing histone acetylation and initial macrophage activation. Thus, GPD2 was proposed to be a critical switch in the time-dependent activation and tolerance of macrophages to LPS stimulation.

The GPD2-ROS relationship also seems to be relevant in rather unrelated tissues, including sperm, placenta, and heart, and even in some tissues where GPD2 activity and expression are low, such as the kidney^105^. During sperm capacitation, GPD2 was reported to be phosphorylated, and its activity was correlated with the acrosome reaction^106^. Using *GPD2* KO mice, it was further shown that sperm capacitation requires GPD2 activity for ROS generation in spermatozoa^107^. The placenta has disproportionately higher GPD2 levels among mitochondrial respiratory enzymes than other tissues^108,109^ and exhibits high GPD2-dependent generation of hydrogen peroxide (H_2_O_2_)^110^, the physiological meaning of which is not clear at this point. More relevant to pathological conditions is probably the role of GPD2-mediated ROS in ischemia‒reperfusion injury (IRI), where resumed blood flow after blood vessel blockage induces paradoxical tissue damage^111^. GPD2-mediated ROS seem to be at least partly responsible for cell death during IRI, which is reduced by miR-210, which inhibits GPD2^112^. In comparison, a protective role of GPD2 in ischemic disease was also reported, where GPD2 deficiency exacerbated cardiac dysfunction during myocardial infarction (MI)^113^. The activation of GPD2 under ischemic conditions, which might result from the increase in intracellular Ca^2+^ in MI, was necessary for ATP synthesis from glycerol as an adaptation to the limited oxygen supply. Another interesting ROS relationship was found in kidney podocytes during diabetic kidney disease^114^, where GPD2 was found to inhibit the receptor-for-advanced-glycation-end-product (RAGE) pathway. RAGE inhibition protected podocytes by enhancing mitochondrial biogenesis/metabolism and lowering ROS, the co-occurrence of which is interesting. Hence, the GPD2-ROS relationship can be either physiological (protective) or pathological (destructive) and, therefore, should be understood in specific contexts.

Many of the roles of GPD2 in pathophysiological conditions have been studied using mice with genetic deletion of *GPD2* (*GPD2* KO). Several different breeds of KO strains (including one liver-specific KO^78^ and another podocyte-specific KO^114^) have been generated, and some of those have been used in multiple studies by different research groups^72,77,84,104^. For example, the mice generated by Eto et al. were used in studies by DosSantos^73^, Ishihama^113^, and Kota^107^ to address phenotypes in different tissues. Additionally, the two tissue-specific KO mice used the same background GPD2^flox/flox^ mice^78,114^. As not all the phenotypes were reproduced in different breeds, it might be important to consider the lineage of the KO breeds. Additionally, some studies have focused on particular tissues, and these tissue-specific phenotypes may need to be further confirmed in tissue-specific KO mice in the future. Furthermore, species considerations may need to be taken when interpreting *GPD2* KO phenotypes. For example, the citrin KO mouse model failed to exhibit symptoms of human citrin deficiency, which required additional *GPD2* KO^115,116^, despite expected changes in some of the molecular metabolic phenotypes^117^. This was suggested to occur due to higher GPD2 activity in the mouse liver than in the human liver, which could have compensated for the phenotypes of suppressed MAS activity in citrin KO mice^115,118^. *GPD2* mutations linked to diabetes^87,88^ and haploinsufficiency of GPD2 in mild mental retardation^119^ are other examples not observed in mouse systems. Overall, these studies demonstrate interesting and complex roles of GPD2 in surprisingly diverse tissues of endocrine, nervous, reproductive, immune, adipose, muscular, and cardiovascular origin.

## Glycerol phosphate dehydrogenases in cancer

**Fig. 2:**
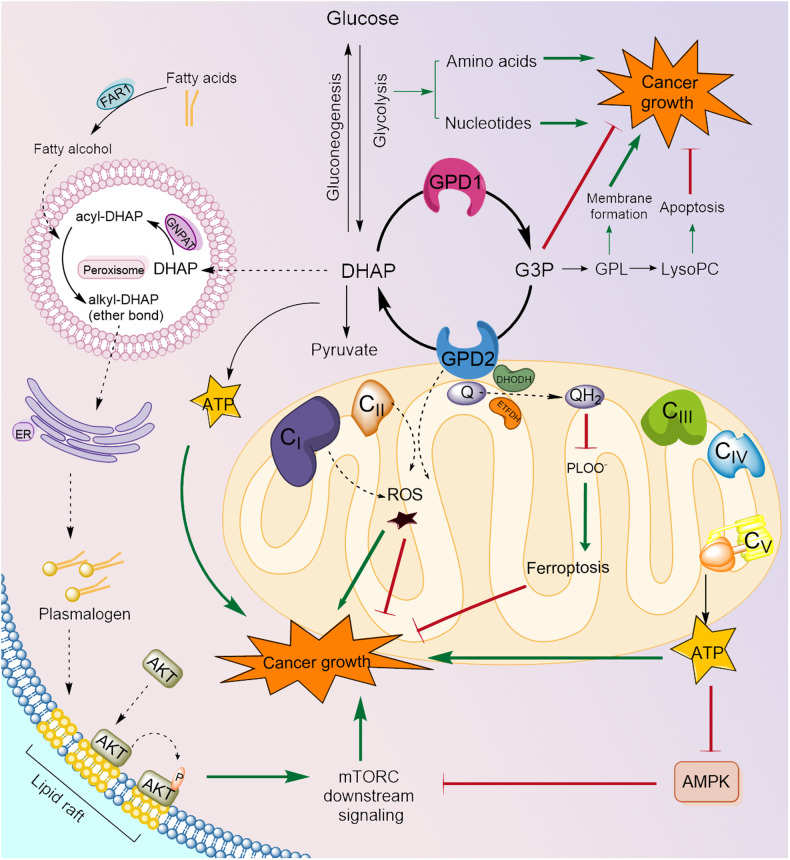
Roles of GPD1 and GPD2 in cancer growth. GPD1 and GPD2 affect cancer cell growth through several mechanisms. GPD1 has mostly tumor-suppressive functions, while GPD2 has mostly tumor-promoting functions, with some studies suggesting otherwise. GPD1 mediates the production of G3P, which has been shown to directly hinder cancer cell growth in some studies or to exert indirect anticancer effects by being a precursor of pro-apoptotic LysoPC. In other cases, G3P formation might enhance cancer growth by contributing to membrane formation through GPLs. Several mechanisms have been reported for the cancer-promoting role of GPD2. GPD2 produces DHAP, which acts, along with fatty alcohols, as an important substrate for ether lipid (plasmalogen) biosynthesis. Ether lipids promote AKT and downstream mTOR signaling by enhancing lipid raft localization and activation of AKT, ultimately supporting cancer growth via a non-bioenergetic mechanism. The bioenergetic contribution of GPD2 to cancer growth has also been reported, wherein it transports reducing equivalents to CoQ, a component of the electron transport chain for ATP generation. Elevated ATP levels may directly fuel cancer cell growth and indirectly impact proliferation by inhibiting AMPK activity and sequentially activating mTOR signaling. The reducing power of GPD2 may also neutralize lipid peroxyl radicals, protecting cancer cells from ferroptosis. Overactivation of GPD2 may trigger reverse electron transport (RET), which results in the production of ROS. Found mostly in immune cells, this RET-driven ROS either activates NF-kB-dependent gene expression or inhibits oxidative metabolism in different cell types. Although not explicitly studied in cancer cells, these immune-related activities may also have implications for cancer cell growth. Dashed arrows and a brown star denote electron transfer and ROS, respectively. See the text for details. AMPK AMP-activated protein kinase, DHAP dihydroxyacetone phosphate, DHODH dihydroorotate dehydrogenase, FAR1 fatty acyl-CoA reductase 1, G3P glycerol 3-phosphate, GNPAT glyceronephosphate O-acyltransferase, GPL glycerophospholipid, PLOO phospholipid hydrogen peroxide radical, Q coenzyme Q/ubiquinone, LysoPC lysophosphatidylcholine, QH_2_ reduced form of coenzyme Q/ubiquinol.

Reprogramming of cellular metabolism is one of the hallmarks of cancer^120,121^. Well-known oncogenes or tumor suppressor genes, such as *KRAS*, *P53*, *MYC*, and *EGFR*, have profound effects on metabolism^122–124^. Additionally, growth factor signaling pathways critical for tumor growth, such as the PI3K-AKT and MEK/ERK signaling pathways, directly engage metabolic pathways through mTOR or c-myc in cancer^125,126^. Furthermore, glycolysis and lipid metabolism are widely altered in cancer^127–129^. Therefore, it should not be surprising that the components of GPS, which are at the crossroads of glycolysis and lipid metabolism, are abnormally modulated in cancer (Fig. 2). Interestingly, GPD1 and GPD2 expression is negatively correlated in most cancer types^130^, even though they are components of the GPS. Supporting this is that the roles of each GPD are mostly opposite in cancer, with GPD1 generally acting as a tumor suppressor and GPD2 acting as a tumor promoter. Individual examples and possible mechanisms for regulating cancer cell proliferation by altering cellular metabolism are presented below.

### GPD1 acts as a tumor suppressor in most cases

It was initially demonstrated that GPD1 activity is markedly lower in malignant tissues of the colon and rectum than in their normal counterparts^131^. Subsequently, consistent studies have reported that *GPD1* expression is significantly reduced in several other cancers, such as breast, lung, bladder, and kidney cancer, and that a low expression level of GPD1 is significantly correlated with a poor survival rate^17,132,133^. Particularly for breast cancer, a proteomics-based study revealed the lowest expression of GPD1 protein in triple-negative breast cancer (TNBC), suggesting a possible relationship between GPD1 and hormone receptors in breast cancer^134^. Interestingly, a decreased GPD1 level was also found in serum samples of TNBC patients, suggesting the possible use of GPD1 as a diagnostic biomarker^135^. In kidney cancer, overexpression of GPD1 downregulated various lipid synthesis genes, leading to a decrease in lipid droplets^17^, the enrichment of which is a characteristic and cancer-requiring feature of ccRCC^136–138^. In addition to these lipid metabolic changes, decreased cell proliferation, migration, and invasion, as well as the inhibition of xenografted tumor growth, were also observed, consistent with the correlation between high GPD1 protein levels and better ccRCC patient survival.

It should also be mentioned that some reports have suggested that GPD1 plays a tumor-promoting role. For example, a significantly higher GPD1 level was found in dormant brain tumor stem cells than in normal neuronal stem cells, which can contribute to differences in glycerophospholipid (GPL) metabolism and tumor relapse after chemotherapy^139^. KO of *GPD1* in these cells downregulated genes related to stem cell identity, cell cycle progression, and the mTOR pathway, as well as the phosphorylation of S6. In another study under hypoxic conditions, GPD1-driven G3P synthesis was shown to maintain 143B cancer cell proliferation^38^, and *GPD1*/*GPD1L* double knockdown (KD) in mouse kidney cancer cells inhibited lipid synthesis and in vitro/in vivo tumor growth^130^. An observational bladder cancer study also suggested correlated increases in GPD1 and fatty acid synthetic enzyme activities in tumor tissues^140^. With more clinical studies showing lower GPD1 in cancer tissues, it seems that the tumor-promoting mechanism of GPD1 may operate in particular contexts. These may include cancer types (e.g., ccRCC vs. non-ccRCC kidney cancer or bladder vs. kidney cancer), species (mouse vs. human), or experimental conditions (e.g., hypoxia, glycolysis, or dual knockdown of *GPD1* and *GPD1L*). The exact extent of these GPD1 tumor-promoting roles and the context may require further studies.

### Mechanistic aspects of GPD1 in cancer cell proliferation

As many studies have demonstrated the low expression of GPD1 in various types of cancer and have suggested that it is a tumor suppressor, most of the mechanistic studies have investigated the effects of GPD1 overexpression on cancer cell proliferation and growth. There seem to be several mechanisms through which it either is regulated or regulates cell survival/growth pathways. In breast cancer cells, Zhou et al. showed that GPD1 is a direct target of a microRNA (miRNA), miR-370, which downregulates it post-transcriptionally^132^. As miR-370 is known to be upregulated in breast cancer cells and related to tumor progression, this study revealed an upstream regulator of GPD1 in breast cancer. Regarding the downstream mechanisms by which GPD1 regulates cancer growth, two studies proposed the common involvement of the reaction product G3P in different contexts. In one study, GPD1 and metformin synergized to increase G3P, which exhibited a direct cancer-inhibitory effect in several cancer types^141^. For the cancer-inhibition mechanism, the authors specifically excluded possible pathways of mTOR or methylglyoxal toxicity by increased G3P and, instead, proposed the inhibition of mitochondrial respiration and ATP generation by G3P. Notably, the increase in G3P induced by GPD1 overexpression (OE) was not statistically significant, but GPD1 OE alone, without metformin, reduced in vivo tumor xenograft growth and mitochondrial oxygen consumption. In another study on bladder cancer^133^, GPD1 OE significantly increased G3P and NAD^+^ levels, as well as that of lysophosphatidylcholine (LysoPC). LysoPC activated its receptor (platelet-activating factor receptor; PAFR)-mediated transient receptor potential vanilloid 2 (TRPV2) channel opening, ultimately leading to Ca^2+^ influx and apoptosis. Therefore, the downstream pathways from G3P in the two studies seemed to diverge to mitochondrial respiration and to membrane channel-mediated Ca^2+^ influx. Future studies should investigate whether these differences are due to different cancer types or whether there are commonalities in these mechanisms. Additionally, the use of several tens or hundreds of millimolar concentrations of G3P for observing cancer cell phenotypes in both studies might need to be considered in future studies for any effect of osmolarity.

However, another study employing the GPD1 OE approach revealed interesting mutual upregulation between HIF1α and GPD1, which affects lipid metabolism in kidney cancer^17^. GPD1 OE suppressed mitochondrial basal respiration and ATP synthesis, activating AMPK and inhibiting mTOR. This, in turn, decreased the levels of lipid droplets, which are primarily composed of neutral triacylglycerol and cholesterol esters^142^, although it increased the total phospholipid content. It would be worthwhile to compare these findings with those of another study on mouse kidney cancer, which suggested apparently different roles for GPD1 in lipid synthesis^130^, as lipid accumulation is a characteristic phenotype in ccRCC^143,144^, the most common type of kidney cancer^145^. Using *GPD1*/*GPD1L* double KD in mouse renal adenocarcinoma Renca cells, Yao et al. observed a decrease in lipid synthesis, particularly GPL, and mitochondrial respiration. From a lipid metabolic perspective, the increased synthesis of phospholipids by cytosolic GPD was identical in both studies, but the characteristic features of ccRCC, the increase in neutral lipids and its role in tumors, seem to have been better addressed in the former study. Yao et al. also suggested that GPL synthesis and redox homeostasis for tumor growth are driven by efficient G3P production by GPD1/GPD1L, which is different from the inhibition of tumor growth by G3P and GPD1 reported by two other studies in different cancers, as stated above^133,141^. The tumor promotion by GPD1, as shown in Yao et al.’s study, was also mechanistically suggested with several models under hypoxia or dysfunctional ETC conditions^38^. *GPD1* KO significantly abolished G3P synthesis and increased the NADH/NAD^+^ ratio when the ETC was inhibited in 143B (osteosarcoma) and HeLa (adenocarcinoma) cells, consistent with all other studies showing G3P generation by GPD1. However, *GPD1* KO increased the sensitivity of cancer cells to the antiproliferative effects of ETC inhibitors and suppressed xenograft tumor growth under ETC inhibition, indicating that GPD1 promoted tumor growth. Moreover, GPD1 OE promoted the activation of GPS to rescue cancer cell proliferation under CI inhibition, demonstrating that GPS can compensate for mitochondrial dysfunction in ATP production and redox homeostasis.

Overall, GPD1-mediated G3P production was consistently observed in all the cited studies employing both the *GPD1* KO/KD and OE approaches. The downstream effects of increased G3P include different mechanisms for the tumor-promoting and tumor-suppressing activities of GPD1. In this respect, the results from the GPD1 OE and *GPD1* KO/KD setups may not necessarily be opposite, as compensation mechanisms may differ and, therefore, should be compared with the particular approach in mind. For the direct effect of G3P, the compound concentration, hence osmolarity, and its permeability across the cell membrane should be carefully considered in future studies. These findings suggest that despite the availability of an atomic-level structure of human GPD1, the functions of GPD1 in cancer have yet to be fully elucidated.

### GPD2 is upregulated in many cancer types

In contrast to the generally lower expression of GPD1 in cancer, an early study showed that patients with hepatocellular carcinoma (HCC) possess higher GPD2 activity than normal controls^146^. Subsequently, GPD2 activity has been shown to be higher than succinate dehydrogenase activity in various cancer types belonging to the amine precursor uptake decarboxylation system^79^. Moreover, *GPD2* expression and activity were higher in prostate and liver cancer cell lines and tissues than in their normal counterparts^147,148^. Additionally, in prostate cancer, a recent study revealed an interesting posttranslational processing of GPD2 in the formation of a functional dimer (see above)^49^. As the proposed processing enzyme IMMP2L, along with GPD2, inhibits senescence^149^, it may be interesting to study whether GPD2 also inhibits oncogene-induced senescence that suppresses cancer initiation and progression^150^.

The effect of GPD2 on drug sensitivity and tumor grade/prognosis was also investigated. The expression of GPD2 was higher in thyroid cancer tissues than in normal thyroid tissues^151^. More importantly, thyroid cancers with higher GPD2 levels and metastatic tumors derived from them responded better to metformin than did those with lower GPD2 levels. Posttranslational modification may be related to GPD2 involvement in tumor grade in glioma^69^. The level of the phosphorylated form of GPD2 at threonine 10 (GPD2 pT10), which has a higher substrate affinity, was higher in high-grade (grade IV) glioblastoma than in grade I/II astrocytoma despite no difference in the unmodified GPD2 levels. Additionally, patients with an above-median GPD2 pT10 status had worse survival than those with a below-median GPD2 status. Like GPD1^139^, GPD2 is related to cancer cell stemness. In the Huh-7 HCC cell line, a subpopulation with the stem cell marker CD133 exhibited increased GPD2 levels^152^ and in vivo tumorigenicity^153^, and KD of *GPD2* decreased anchorage-independent cell proliferation^152^. An analogous involvement of GPD2 in neuronal cancer cells was reported by the same group^154^. A broader investigation of the expression of GPD2 revealed that its level is higher in tumors than in normal tissues in most cancer types and that higher GPD2 expression is correlated with poorer survival in some cancers^155^. Notably, when all the cancer types were combined, the expression levels were higher in tumor tissues, and higher GPD2 levels were correlated with poorer survival.

Not surprisingly, there may be some cases in which the above cancer-promoting roles do not apply. Highlighting the anti-correlation of GPD1 and GPD2 in cancer, a recent study showed that GPD1 has tumor-promoting effects and that GPD2 has tumor-suppressing effects^130^. They showed that *GPD2* KD in mouse renal adenocarcinoma Renca cells enhanced tumorigenicity in the syngeneic graft setting, which was recapitulated in human ccRCC cell lines (786-O and Caki-1) in vitro. Another study reported that GPD2 inhibits melanoma metastasis in vitro and in vivo through downregulation of NRF2^156^. Interestingly, NRF2 also has well-known dual roles in tumor initiation, progression, and chemotherapy^157^. Overall, GPD2 seems to have generally tumor-promoting effects, with higher expression in tumor tissues. Nevertheless, as in the GPD1 case, there can be some specific contexts where GPD2 may have tumor-suppressive roles, and the extent of these effects should be addressed in future studies.

### GPD2 regulates cancer progression via various mechanisms

As stated above, there are still apparent discrepancies in the role of GPD2 in cancer, and therefore, it is crucial to study the detailed mechanism by which GPD2 regulates cancer growth. Along with the increased activity of GPD2 in prostate cancer cells, prostate cancer cells produce 2- to 3-fold more H_2_O_2_^147^ and express higher levels of antioxidant enzymes, including catalase, MnSOD, and CuZnSOD, than normal prostate epithelial cells^148^. COX levels were low in a subset of the cell lines. Increased ROS are observed in many cancers^158^, and ROS can cause DNA mutations that can initiate carcinogenesis^159^. Nevertheless, to protect against too much damage, cancer cells also engage in detoxifying mechanisms^160^. As GPD2-specific superoxide production is comparable to that at other major production sites in mitochondria^161^ and G3P can be a significant contributor to cellular H_2_O_2_^162^, the above results in prostate cancer seem to point toward GPD2-driven ROS in cancer. Despite evidence of GPD2-driven ROS generation in different tissues and its roles in tissue functions^105,161,163^, its contribution to cancer still requires more evidence in terms of the involvement of different ROS forms and regulators of GPD2-driven ROS generation. Additionally, how ROS is increased may need to be considered, as the glycolysis-driven increase in ROS reported in prostate cancer^148^ contradicts the observation that prostate cancer cells tend to have less glycolytic flux than normal prostate cells^49,164^.

In other cancers, enhanced glycolytic flux is a well-established phenomenon (Warburg effect), and the ensuing bioenergetic metabolism is known to be important for cancer cell growth^127,129^. In fact, the physiological relevance of GPD2 has been ascribed to its involvement in bioenergetics through glucose metabolism. In cancer, GPD2-driven bioenergetic mechanisms involving glucose have also been described in several cancer types. For example, a decrease in G3P in human liver cancer tissue compared to normal tissue was described with increased glycolytic flux^165^, suggesting increased GPD2 activity. Enhanced GPD2-mediated G3P consumption might occur in para-preneoplastic hepatocytes, rather than in preneoplastic cells themselves, to supply glucose in the early stage of rat liver cancer^166^. In glioma, macrophage-derived IL-1β induces the activation of GPD2 through interaction with PKCδ, ultimately leading to enhanced glycolysis and proliferation^69^. Although not explicitly stated, an increase in the DHAP/G3P ratio due to the activation of GPD2 and hence a decrease in glycolysis may contribute to increased glycolytic flux, as measured by ^13^C-lactate generation from ^13^C-glucose. Among the available studies, studies on the effect of metformin on thyroid cancer have focused on the bioenergetic contribution of GPD2 to cancer growth^151^. In line with the finding that GPD2 is a target of metformin in gluconeogenesis^167,168^, metformin was shown to lower GPD2 expression for its suppressive activity on thyroid cancer. This inhibitory activity resulted in decreased oxidative phosphorylation (OXPHOS), leading to decreased growth of thyroid cancer cells. In further support of the bioenergetic role of GPD2, the overexpression of GPD2 in thyroid cancer cells increased mitochondrial respiration and ATP production, resulting in increased cell growth. They also showed that metformin inhibited the metastasis of thyroid cancer cells with high GPD2 and OXPHOS levels but not those with lower GPD2 and OXPHOS levels. The potential of GPD2 as a target of metformin for its anticancer activity is also notable considering that both CI-AMPK-mTOR pathway modulation and IGF pathway modulation through lower blood insulin have been conventionally suggested to explain the mechanism underlying the anticancer activity of metformin^169,170^. The involvement of GPD2-mediated oxidative metabolism, e.g., oxygen consumption and ROS production, has also been described in prostate cancer cells compared to normal epithelial prostate cells^49^. Both fully fledged cancer cells and cancer stem cells may depend on GPD2-mediated ATP synthesis for sphere formation and growth, as shown for liver and neuroblastoma cancer cells by the same authors^152,154^.

Although the ROS or bioenergetic mechanism of GPD2 in cancer growth is related to well-established functions of GPD2 in normal physiology, recent reports have studied less-explored aspects of GPD2-mediated metabolism for cancer growth or survival: ether lipids or lipid peroxidation. Although GPD2 is considered the crossroad of glucose and lipid metabolism, its role is mainly attributed to G3P, the substrate of GPD2 and the rate-limiting metabolite in glycerolipid synthesis^171,172^. In comparison, DHAP is the product of the GPD2-mediated reaction and the starting substrate for ether-linked lipids^173–175^. With 4T1 mouse breast cancer cells, it was shown that GPD2 is causally involved in tumorigenesis both in vitro and in vivo^155^. Importantly, it was not GPD2-mediated bioenergetics, e.g., respiration or ATP generation, but GPD2-derived DHAP and the resulting ether lipid biosynthesis that were critical for the cancer growth. Further mechanistic investigation revealed the role of the GPD2-DHAP-ether lipid-AKT axis in tumor cell growth. Another study related to lipids suggested that GPD2-mediated reduction of CoQ to CoQH_2_ protects against mitochondrial lipid peroxidation, preventing ferroptosis in cancer cells both in vitro and in vivo^176^. Interestingly, they showed that G3P supplementation can rescue RSL3-induced cell death in a GPD2-dependent manner. Nevertheless, the contribution of GPD2 to CoQ reduction seems smaller than that of DHODH.

Overall, several mechanisms may influence the role of GPD2 in cancer cell growth. Among them, ROS and bioenergetics, along with CoQ reduction, are connected with GPD2’s established role in the mitochondrial ETC. DHAP-mediated ether lipid synthesis and its activation of the AKT-mTOR pathway seem to be the only route that does not directly involve the mitochondrial electron transport mechanism. Additionally, this DHAP-related mechanism seems to be the first by which GPD2 modulates one of the most altered signaling pathways in cancer, the PI3K/AKT/mTOR pathway. As many metabolic enzymes have been shown to have non-metabolic roles, e.g., functioning as transcription factors and signaling molecules^177–179^, future research may add additional roles to the list.

### Bioenergetic contribution of GPD2

As a well-recognized function, GPD2 transfers reducing equivalents to the ETC, specifically to complex III (CIII), it may be worth discussing the bioenergetic contributions of GPD2 in both cancer and non-cancer contexts. In addition to CI and complex II (CII), additional proteins, such as electron transfer flavoprotein dehydrogenase and dihydroorotate dehydrogenase (DHODH), are known to transport electrons to CIII. Although detailed studies comparing the exact contributions of each of these components have been scarce, the involvement of ETC components other than CI and CII seems to be substantial only under some specific conditions rather than being universally present. For instance, the deletion of GPD2 in mouse bone marrow-derived macrophages had minimal effects on basal and maximal respiration but significantly decreased the oxygen consumption rate (OCR) upon short-term LPS stimulation^104^. Interestingly, after long-term LPS stimulation, which induces tolerance, the LPS-mediated decrease in the OCR in the WT was attenuated in the GPD2-deleted cells, suggesting a negative effect of GPD2 on oxygen consumption under these LPS-tolerant conditions. Similarly, Bajzikova et al. reported that the contribution of DHODH to oxygen consumption was less than 10% of total respiration (sum of CI, CII, and DHODH) and that *DHODH* KO did not affect overall respiration in murine mammary carcinoma 4T1 cells^180^. A similar quantitative contribution of GPD2 to overall cellular respiration was reported in the same 4T1 cells^155^. Additionally, several lines of evidence revealed only minor contributions of GPD2 to the mitochondrial bioenergetics of cancer cells, such as in mouse kidney cancer cells (Renca)^130^ or in human kidney cancer cells, including Caki1 and 769P cell lines^17^. On the other hand, notable contributions have also been reported under other conditions^38,49,176^. For example, a study focusing on metabolism under CI-compromised conditions showed that GPD2 was responsible for the majority of CI-independent OCRs in cancer cell lines that generally have high CI-independent OCRs, such as OVCAR4^38^. In contrast, *GPD*2 KO had no such effect on cell lines with a low CI-independent OCR. Nevertheless, it should be noted that the effect of *GPD2* KO on basal respiration in this study was only modest (~12%) and that the CI-dependent respiration of many cancer cells, as measured with piericidin A, was much larger than the CI-independent respiration, which includes GPD2-dependent respiration. In another study with prostate cancer cell lines, cancer cells exhibited a higher OCR than did normal epithelial cells, which could be associated with a higher presence of GPD2, despite the equal levels of other ETC components, such as CI through complex IV (CIV)^49^. A substantial decrease in the basal OCR (~40%) in HCT116 cells upon *GPD2* KO, accompanied by an increase in the CoQ/CoQH_2_ ratio, was also reported^176^, although this might be at odds with the very little CI-independent respiration (~5% of the CI-dependent respiration) reported by Liu et al. in the same cells^38^. This finding, along with further experiments, indicated the involvement of GPD2 in the ferroptosis defense mechanism in cancer cells, specifically through the CoQ system, suggesting the bioenergetic contribution of GPD2. Although DHODH was also proposed to have a similar ferroptosis defense function^181^, it is interesting that the overexpression of DHODH completely recovered ferroptosis sensitization in *GPD2* KO cells, whereas the overexpression of GPD2 in *DHODH* KO cells only partially rescued the ferroptosis sensitization phenotype. These data suggest that GPD2 plays a narrower role in regulating ferroptosis than DHODH. Taken together, the overall contribution of GPD2 to cellular respiration seems to be minimal to modest but may be important under specific conditions, such as in prostate cells, during ferroptosis, or under CI-compromised conditions. Other enzymes, such as DHODH, may play similar roles under these conditions, which warrant further investigation.

## Therapeutic targeting of GPD1 and GPD2 in cancer

Compared to other components of the bioenergetic machinery, e.g., the TCA cycle, glycolysis, and the ETC, much less is known about the roles of GPS or its components GPD1 and GPD2 in cancer. Therefore, the number of modulators targeting GPD1 and GPD2 is relatively small but holds high potential given the wealth of new studies mentioned above.

For GPD1, no inhibitor studies have been reported, probably because it is known for its tumor-suppressive activity. Interestingly, an activator of GPD1, wedelolactone, exhibited anticancer activity. In bladder cancer, the activation of GPD1 by wedelolactone successfully decreased cancer cell viability by ~50% and reduced tumor weight by ~70% in xenograft models^133^. As this natural product also has other activities, further studies are needed to understand the contribution of wedelolactone-mediated GPD1 activation to its anticancer activity.

Currently, there are only a few inhibitors specifically developed for GPD2; the iGP series^182^ and KM04416^183^ were identified from small molecule screening. KM04416, with an isothiazolone moiety, was discovered based on its ability to inhibit H_2_O_2_ generation from G3P (with an EC_50_ of ~1 μM) and prostate cancer cell growth. The iGP-series containing the benzimidazole moiety was also discovered by G3P-based H_2_O_2_ generation screening. Among those, iGP-1 and iGP-5 inhibited actual GPD2 activity (G3P-mediated reduction in DCPIP), and the latter exhibited an order of magnitude higher activity (Ki ~1 μM). Additionally, iGP-1 exhibited negligible activity on GPD1. As these compounds were derived from a single-round screening, further medicinal chemistry-based optimization may lead to more potent inhibitors. It is also worth noting that the activity of iGP-1 may not be achievable under all experimental conditions, as it neither increased the G3P level nor sensitized colon cancer HCT116 cells to ferroptosis, which was observed in *GPD2* KO cells^176^. Moreover, iGP-1 did not suppress the growth of cancer cells in our hands either (data not shown), whereas KM04416 did^155^. This may be due to the use of different cell lines and the fact that the iGP series were tested for GPD2 activity per se but not for its anticancer activity. Generally, the potencies of both the KM04416 and iGP series are in the medium range, and more studies are needed for inhibitors with stronger specificity and potency.

Although it was not specifically developed as a GPD2 inhibitor, metformin is a well-known antidiabetic drug that has been shown to inhibit GPD2 in two ways. First, in diabetes, metformin was shown to inhibit GPD2 non-competitively, reducing the conversion of G3P to DHAP and changing the cellular redox state, which ultimately lowered the gluconeogenic flux from glycerol to glucose^167,168^. Second, metformin was shown to lower the expression of GPD2 and thus OXPHOS activity in thyroid cancer, which was responsible for its antithyroid cancer effect^151^. Notably, 50 μM metformin inhibited recombinant GPD2 and mitochondrial oxygen consumption in gluconeogenesis studies^167^; this dose was much lower than the concentrations required for metformin to exhibit in vitro anticancer activity or CI inhibition (≥1 mM^184–186^), as used in thyroid cancer studies. Interestingly, metformin exhibited an enhanced anticancer effect in the GPD1-overexpressing background^141^. An in vivo synergistic effect between GPD1 and metformin was observed even though the G3P level was not significantly enhanced by GPD1 overexpression in vitro. Therefore, synergistic effects may occur regardless of the somewhat controversial supraphysiological G3P concentrations used to address the mechanism involved. Additionally, it would be interesting to determine whether patients with high GPD1 and/or GPD2 levels in their tumor tissues might respond better to metformin treatment. In future mechanistic studies, it may be worth noting that metformin inhibits only GPD2 without altering the activity of GPS or GPD1^167^.

Other molecules with GPD2-inhibiting activity have been reported, including several from natural product sources. As such, these molecules may not be specific to GPD2 but are expected to affect other enzymes. α-tocopheryl succinate, a mitochondrial CII inhibitor, was found to inhibit GPD2 more efficiently than CII in terms of substrate-mediated oxygen consumption (IC_50_ of ~10 μM) and H_2_O_2_ generation^187^. For natural products, scopolin, esculetin, and taraxasterol inhibited tumor growth by suppressing GPD2-related glycolysis^188–190^. Scopolin and esculin were shown to bind to GPD2 using the monolith nanotemp fluorescence method, but there were significant discrepancies in concentrations for GPD2 inhibition and cell phenotype inhibition, which may require caution in interpreting the results. Taraxasterol inhibited GPD2 inhibition at 15 μM and its apoptosis-inducing effect could be partially reversed by overexpression of GPD2.

## Conclusions and future perspectives

GPD1 and GPD2 have been implicated in three major processes: glucose metabolism (glycolysis and gluconeogenesis), bioenergetics (NAD^+^ recycling, ATP production, and ROS generation), and lipid metabolism (G3P-derived glycerolipids and DHAP-derived ether lipids). These processes are interrelated, and GPS component enzymes are at key crossroads between energy-consuming and energy-generating pathways. Due to their key roles in metabolism, alterations in GPD activity have been associated with various (patho)physiological conditions, such as diabetes, obesity, muscle regeneration, brain neurotransmission, immune regulation, and cancer, and the causative mechanisms involved are being investigated. Nevertheless, there are some apparent discrepancies among studies, including but not limited to, the pro- vs. anti-tumorigenic roles of GPD. The differences might be context-dependent and might not have been clearly defined, such as cancer types, or due to the limitations of the methodological approaches, e.g., supraphysiological concentrations of G3P.

There are also several points that future research should address based on recent advancements in the knowledge of GPDs. First, more studies are needed to explore the non-conventional or non-bioenergetic roles of GPD2, such as ether lipid-related functions. This is becoming an important issue with three recent independent reports on the absence of ATP production and/or changes in basal oxygen consumption in *GPD2* KO or KD systems^78,130,155^. Therefore, it will be interesting to explore whether the ether lipid synthesis observed in cancer is also relevant in other systems, including muscle, adipose tissues, or immune cells, in which GPD is reported to play important roles. Additionally, the role of GPD1 in ether lipid synthesis should be an interesting topic given that it consumes DHAP. Second, the role of the GPS component as a signaling molecule should be explored. A recent study on a cell line suggested that DHAP activates mTORC1 signaling independently of energy stress or growth factor signaling^191^. Due to the importance of mTORC1 in cell survival and growth, studies in other cells or in vivo systems are highly anticipated. Additionally, GPD1 was shown to affect HIF1α levels in glioblastoma. HIF1α signaling is also important in many different systems, and therefore, the GPD1-HIF1α relationship should be studied in many other cancers and non-cancer systems. These signaling roles of GPD-related metabolites may be another example of metabolic enzymes’ second function (moonlighting). Third, as GPDs are enzymes, specific inhibitors are needed for both mechanistic studies and practical use. Currently available inhibitors are not satisfactory in terms of potency and specificity. The activator of GPD1 may also be relevant because it is known to be involved in tumor suppression. Fourth, the reported negative correlation between GPD1 and GPD2 is intriguing from both mechanistic and functional perspectives. The two components of the GPS are expected to correlate with each other, but they do not in cancer. A possible mechanism was proposed in ccRCC, where GPD1-mediated stabilization of HIF1α transcriptionally represses GPD2 expression, which resulted in tumor suppression^17^. Despite showing the same negative correlation, another study in mouse kidney cancer cells showed GPD1-mediated lipid synthesis and tumor growth^130^. Therefore, the functional consequences of these negative correlations need to be clarified. Additionally, the existence of any mechanistic factors other than HIF1α and the (patho)physiological contexts in which these factors are involved, other than kidney cancer, should be investigated. Notably, increased and decreased activities of GPD1 and GPD2, respectively, in all tissues of the jerboa during hibernation have been reported, except skeletal muscles for GPD2^192^, thus indicating the existence of other conditions for the negative correlation.

Overall, decades of research on GPS have provided insights into the functions of its components across diverse conditions, and future studies are expected to address important mechanistic and functional questions.
